# Electrospun Nanofiber Composites for Drug Delivery: A Review on Current Progresses

**DOI:** 10.3390/polym14183725

**Published:** 2022-09-07

**Authors:** Renatha Jiffrin, Saiful Izwan Abd Razak, Mohamad Ikhwan Jamaludin, Amir Syahir Amir Hamzah, Muadz Ahmad Mazian, Muhammad Azan Tamar Jaya, Mohammed Z. Nasrullah, Mohammed Majrashi, Abdulrahman Theyab, Ahmed A. Aldarmahi, Zuhier Awan, Mohamed M. Abdel-Daim, Abul Kalam Azad

**Affiliations:** 1 Bioinspired Device and Tissue Engineering Research Group, School of Biomedical Engineering and Health Sciences, Faculty of Engineering, Universiti Teknologi Malaysia, Skudai 81300, Johor, Malaysia; 2 Sports Innovation & Technology Center, Institute of Human Centered Engineering, Universiti Teknologi Malaysia, Skudai 81300, Johor, Malaysia; 3 Nanobiotechnology Research Group, Department of Biochemistry, Faculty of Biotechnology and Biomolecular Sciences, Universiti Putra Malaysia, Serdang 43400, Selangor, Malaysia; 4 Faculty of Applied Science, Universiti Teknologi MARA, Cawangan Negeri Sembilan, Kampus Kuala Pilah, Kuala Pilah 72000, Negeri Sembilan, Malaysia; 5 Kolej GENIUS Insan, Universiti Sains Islam Malaysia, Nilai 71800, Negeri Sembilan, Malaysia; 6 Department of Pharmacology and Toxicology, Faculty of Pharmacy, King Abdulaziz University, Jeddah 21589, Saudi Arabia; 7 Department of Pharmacology, Faculty of Medicine, University of Jeddah, Jeddah 23881, Saudi Arabia; 8 Department of Laboratory & Blood Bank, Security Forces Hospital, P.O. Box 14799, Mecca 21955, Saudi Arabia; 9 College of Medicine, Al-Faisal University, P.O. Box 50927, Riyadh 11533, Saudi Arabia; 10 Basic Science Department, College of Science and Health Professions, King Saud bin Abdulaziz University for Health Sciences, National Guard-Health Affairs, P.O. Box 9515, Jeddah 21423, Saudi Arabia; 11 Department of Clinical Biochemistry, Faculty of Medicine, King Abdulaziz University, Jeddah 21589, Saudi Arabia; 12 Department of Pharmaceutical Sciences, Pharmacy Program, Batterjee Medical College, P.O. Box 6231, Jeddah 21442, Saudi Arabia; 13 Pharmacology Department, Faculty of Veterinary Medicine, Suez Canal University, Ismailia 41522, Egypt; 14 Faculty of Pharmacy, MAHSA University, Bandar Saujana Putra, Jenjarom 42610, Selangor, Malaysia

**Keywords:** electrospinning, nanofiber, drug delivery, drug release, nanofiber composite

## Abstract

A medication’s approximate release profile should be sustained in order to generate the desired therapeutic effect. The drug’s release site, duration, and rate must all be adjusted to the drug’s therapeutic aim. However, when designing drug delivery systems, this may be a considerable hurdle. Electrospinning is a promising method of creating a nanofibrous membrane since it enables drugs to be placed in the nanofiber composite and released over time. Nanofiber composites designed through electrospinning for drug release purposes are commonly constructed of simple structures. This nanofiber composite produces matrices with nanoscale fiber structure, large surface area to volume ratio, and a high porosity with small pore size. The nanofiber composite’s large surface area to volume ratio can aid with cell binding and multiplication, drug loading, and mass transfer processes. The nanofiber composite acts as a container for drugs that can be customized to a wide range of drug release kinetics. Drugs may be electrospun after being dissolved or dispersed in the polymer solution, or they can be physically or chemically bound to the nanofiber surface. The composition and internal structure of the nanofibers are crucial for medicine release patterns.

## 1. Introduction

Nanostructured materials, also known as nanomaterials, are becoming more common in our daily lives and are considered as the trendiest basic materials. Nanomaterials also offer incredible promises for enhancing the performance of existing materials while at the same time introducing new features and uses. Nanomaterials have sparked tremendous interest in research and industrial applications during the last few decades. Nanomaterials, defined by their size within the nanoscale, usually 1 to 100 nm, are of significant interest due to their limitless potential application in the health care area [1]. Nanomaterials have gained a lot of interest due to their distinct features such as a huge surface area and designed properties such as high porosity [2]. At the nanoscale, these nanoparticles offer a number of ways to combine materials in new ways by taking advantage of the unique way these materials interact with each other. [2]. Numerous studies have been conducted using biomaterials with a definite 3D structure and cell-informative signals with components similar to the extracellular matrix (ECM) to control the cycles’ biological activity [3,4,5]. Many ECM molecules contain a diversity of intertwined nanoscale fibrous constructions that promote cell adherence and bioactivity, therefore producing architectural scaffolds that imitate ECM [6]. Nanomaterials are now being researched in various disciplines including self-assembly and thin films, quantum dots, nanofibers, nanorods, nanotubes, nanowires, nanocrystals, and nanofoams [7]. It is widely accepted that nanofibers are one of the most fascinating and significant 1D nanostructures that may be employed in nonwoven membranes.

**Figure 1:**
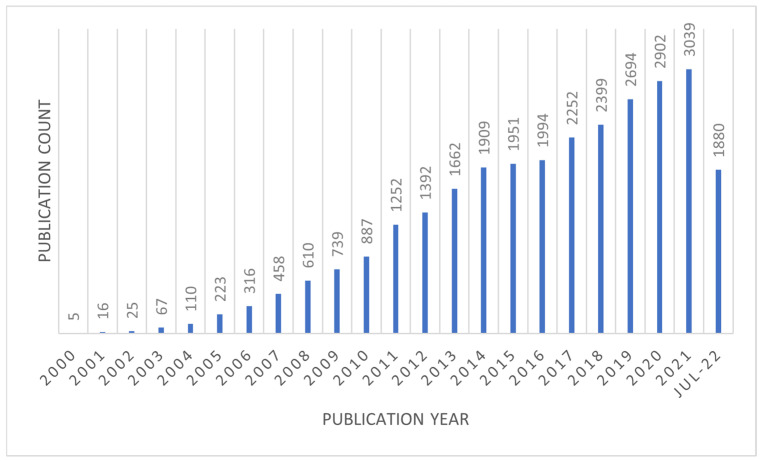
The annual value of scientific publications correlated to electrospinning for the past 22 years. [Data analysis of articles was done using Scopus (The premier source for scientific, technological, and medical research worldwide)] as of 7 July 2022, using the keywords “Electrospinning nanofiber”.

Scaffolds with a nanofibrous structure have been created via phase separation, self-assembly, and electrospinning [8]. Among the processes to produce nanofibrous scaffolds, the electrospinning approach has received much attention from numerous industries. Nanofiber membranes are created using electrospinning, also known as electrostatic spinning. This technique is a unique and basic method that is easy to use, cost-efficient, and has the potential for upscaling, allowing for new industrial applications [9,10]. Recent research and commercial interest in electrospinning, a widely used process for electrostatic fiber production that harnesses electrical forces to make polymer fibers with diameters ranging from 2 nm to several micrometers, has increased dramatically [11,12]. Worldwide research and publications linked to electrospinning have gradually increased over the last decade. The data in Figure 1 demonstrates that over the past 22 years, the total number of publications in electrospinning have elevated remarkably from only five papers in 2000 to 1880 in July 2022. These data were retrieved from Scopus using the term electrospinning nanofibers and covered a variety of topics including improvements in electrospun functional nanofibers [13,14,15,16], electrospinning processing parameters [17,18], and electrospun characterization for a variety of applications [19,20,21,22].

Nanofibers excite a lot of interest nowadays due to their outstanding characteristics. Nanofibers are fibers with diameters ranging from 1 to 100 nanometers. Nanofibrous materials are being researched and created because they hold great potential for a wide range of uses while also achieving some of the benefits of nanostructured materials. Moreover, the field of nanofibers has piqued the attention of many in the fields of biotechnology and medicine, and it has seen rapid progress in recent years. Nanofibers have beneficial properties such as a large surface area-to-mass ratio, adjustable size, shape, and the capacity to construct a porous mesh, which provides an excellent three-dimensional (3D) network environment, which accounts for their increased capabilities [23]. 3D electrospun scaffolds are also helpful for infusion nutrients and cell penetration into the fiber deepening structure [24]. Significant technological developments in the electrospinning technique have allowed for the development and fabrication of desirable features of novel polymeric materials including the structural modification of nanofibers and their capacity to alter wettability, conductivity, and antimicrobial properties [25].

A medication needs a proper drug delivery mechanism to produce the requisite therapeutic effect to ensure its specific release profile. As precisely as feasible, the disposition, time, and release rate of a medication must be adapted to the therapeutic goal of the medicine. It is widely utilized to regulate the medication supply from hydrophilic and biodegradable polymers in health care due to the obvious distinctive traits of nanofibers. A wide variety of medicines such as water-insoluble medications, soluble in water drugs, weakly soluble in water drugs, and macromolecules including bioactive proteins and DNA should be supplied with nanofibers [26]. A composite material is a mixture of two or more different materials with distinct physical and chemical characteristics. When the two materials are combined, they form a material that is tougher, or lightweight. The combination can also increase the strength and rigidity. Biocompatibility, biodegradability, excellent specific modulus, and durability are only a few of the benefits offered by fiber-reinforced composite fibers to the biomedical field [27]. A polymer composite is a multi-phase material that combines reinforcing fillers with a polymer matrix to provide synergistic mechanical qualities that neither component could attain alone [28]. Many studies have been conducted considering the employment of nanofiber composite scaffolds in nerve tissue engineering, antimicrobial applications, blood vessel graft, cancer nanomedicine delivery, soft tissue reconstruction, diabetic wound healing, artificial muscled design, and bone regeneration [29,30,31,32,33,34,35,36]. Ergo, this review article has emphasized the significant ability of electrospinning and post-treatment modification to produce nanofiber composites as drug carriers in drug delivery applications. First, a brief overview emphasizes the electrospinning technique as an approach to fabricate nanofibrous scaffolds for drug delivery purposes. Parameters affecting the fabrication of nanofibers, synthetic and natural polymer nanofiber, nanofiber system type, and drug release mechanism are also topics that are discussed in this review. In addition, this review also highlights the benefits and drawbacks of each material, type, the properties, and characterization approaches of the nanofiber composites utilized in the manufacturing of nanofiber composite scaffolds. Moreover, this review accentuated the latest application of electrospun nanofibers as drug carriers in pharmaceuticals, bone tissue engineering, nerve tissue engineering, periodontal tissue engineering, wound dressing, and cancer therapeutics drug delivery.

## 2. Electrospun Nanofiber

Electrospinning is a technique that employs nanoscale fibers to construct an impermeable nonwoven fabric by driving a liquid jet with a millimeter diameter through an electric field-induced nozzle, which results in the formation of submicron fibers. Generally, electrospinning is an electrohydrodynamic technique. In this process, a liquid droplet is electrified to create a jet, which is then stretched and elongated to produce fibers. During the electrospinning process, a high voltage power supply is commonly applied to the solution, which later causes the production of pendant droplets [37]. The pendant droplet is a result of surface tension when the liquid is discharged from the spinneret [38]. The charging effect on the surface of the pendant droplet created at the tip of the blunt needle is a result of an electric field that causes a wobbliness that changes the shape of the hemispherical droplet into a cone, usually called the Taylor cone [39].

In this process, the repulsive electric forces surpass the surface tension when the applied electric field achieves a threshold value [40]. When the field strength is adequately strong, a jet of liquid is continually extruded from the tip of the cone and shatters, producing charged particles. A steady, continuous stream of charged particles is conceivably constructed in this cone-jet way of operating [41]. The jet loses solvent through evaporation as it travels toward the collector during electrospinning. The diameter, morphology, and characteristics of the final solidified nanofibers are determined solely by the fast evaporation of the solvent, followed by stretching of the jet due to electric forces and jet instabilities [42]. Thinning of the jet allows for an increased surface area per unit volume to ensure aid for evaporation, where this process thins the jet even further, resulting in thinner fibers [43]. As the solvent evaporates, the diameter of the jet shrinks dramatically as the jet solidifies, resulting in ultrafine-diameter fibers [44].

**Figure 2:**
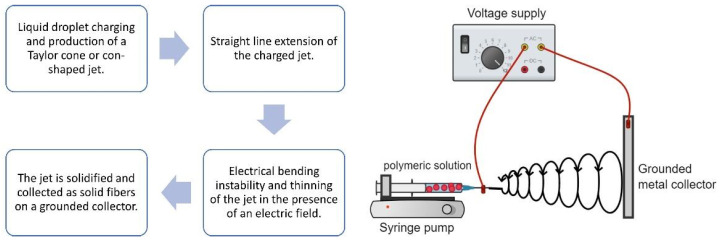
The electrospinning process and schematic setup of the electrospinning.

**Figure 3:**
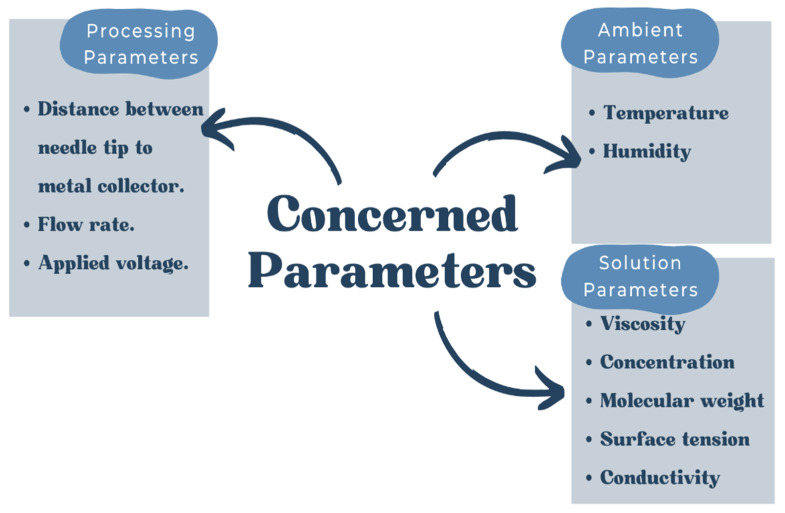
The essential parameters for the electrospinning process.

Figure 2 illustrates the fundamental arrangement for electrospinning, which is fairly simple. A high-voltage power source, a syringe pump, a syringe with a needle with a blunt tip, and a grounded conductive metal collector are the main components of the electrospinning. Although the setup is basic, understanding the concepts and factors that govern the electrospinning process is required before any polymer solutions may be turned into desirable nanofibers. Furthermore, by changing the electrospinning settings, the choice of materials and postprocessing treatments as well as the characteristics of nanofibers may be adjusted to meet specific requirements in terms of the layer thickness, fiber diameter, porosity, and other capabilities [45]. Electrospinning parameters, for instance, processing, solution, and ambient parameters, influence the diameter and structure of the fibers generated [46,47,48,49]. The processing parameters cover the applied voltage, flow rate, or feeding rate and distance between the needle tip to the metal collector. Solution parameters include the viscosity, concentration, molecular weight, surface tension, and conductivity. The ambient or environmental parameters usually include humidity and temperature. Figure 3 illustrates the essential parameters that concern the desirable fabrication of nanofibers.

Can-Herrera et al. [50] conducted an investigation to study the morphological properties of electrospun polycaprolactone (PCL) nanofibers in relation to the applied voltage. According to their research, the fibers had a uniform appearance, and beads were absent at any of the tested voltage levels. When the voltage was increased, large pores and branch-shaped fibers were detected. This may be described in the following way: Multiple jets of electrospinning are induced by high voltages, lowering the electrostatic forces, and stretching the nanofibers [51], due to which shortened fibers are generated. When the voltage was increased from 15 kV to 20 kV, the fiber diameter increased. Increased voltages accelerated the jet toward the collector, resulting in a shorter flight time for stretching the jet preparatory to deposition, allowing for the formation of fibers with a larger diameter [52]. In another study conducted by Bakar et al. [53] on electrospun polyacrylonitrile (PAN) nanofibers, they discovered that the fiber diameters increased as the applied voltage increased. Meanwhile, in research on bubble electrospinning by Liu et al. [54], they discovered that the number of beads present on the nanofibers decreased as the applied voltage increased. Additionally, the increase in the average diameter of fibers may be due to the fact that a higher applied voltage results in a greater electrostatic force, so those with larger diameters that may be unavailable at a low electrostatic force [54].

A study conducted by Zargham et al. [55] on the effects of flow rate on the morphology and deposition of electrospun Nylon 6 nanofibers identified that the flow rate impacted the distribution of the fiber diameters, droplet size, and form at the capillary tip, jet trajectory, Taylor cone retention, regional density, and nanofiber structure. The flow rate fluctuations had an effect on the distribution of the fiber sizes. Clearly, as the flow rate increased, the diameter dispersion of the fibers became broader. To generate continuous fibers, a stable Taylor cone must be formed [55,56]. In addition, in research completed on the effect of the flow rate on poly(vinylidene fluoride) (PVDF) nanofibers by Zulfikar et al. [57], they found that as the flow rate increased, the electrospun fibers generated retained their basic cylindrical form, but the number of bead defects in the fiber mat decreased noticeably. When the flow rate was increased, the fibers developed quicker, resulting in additional strain stress on the grounded collection and the beads not having enough time to form [57]. More solution is expelled from the needle tip in a given amount of time when the feeding rate is increased in which the surface tension may be responsible for forming the beads when the electric field force is insufficient to stretch the jet [58]. Al-Hazeem [59] studied the effect of the distance tip-to-collector on titanium dioxide (TiO_2_) incorporated polyvinylpyrrolidone (PVP) nanofibers. Al-Hazeem [59] identified that the fiber diameter and structure were determined by the distance, as increasing the distance improved the morphology with a smaller average diameter. When the distance was extended, the morphology was enhanced as the diameter decreased; this happened because as the distance was short, there was insufficient time to evaporate the solvent before the deposition of the fibers on the collector, so the fibers may combine. The distance of the tip-to-collector should be suitable to enable sufficient time for the solution to evaporate and stretch before settling on the collector [60]. The distance of 15 cm produced the best outcomes among the chosen distances, with the fiber forming on a regular basis and having an average diameter smaller than the other distances. Bakar et al. [61] discovered that the characteristics of the electrospun polyacrylonitrile (PAN) fibers generated were discovered to be highly dependent on electrospinning parameters such as the PAN solution concentration. The nanofiber diameter increase with the polymer solution concentration was due to the number of macromolecular chains and chain entanglements, which rose with an increasing concentration in the electrospinning fluid [61].

The electrospinning solution viscosity can be enhanced by increasing the concentration of the polymer solution [62]. Nezerati et al. [63] identified that beaded fibers formed at the lowest viscosity of poly(carbonate urethane) (PCU) of 7 Pa·s, uniform fibers formed at an intermediary viscosity of 13 Pa·s, whilst at a higher viscosity of 23 Pa·s, the diameter of the fiber increased. The formation of beads was due to the solution at a lower concentration lacking the viscosity required to withstand fiber deformation without fault under the applied electric field [63]. Meanwhile, a higher viscosity of the high concentration of PCU produced greater viscoelastic forces, which opposed the axial stretching during whipping in electrospinning, generating a larger nanofiber diameter [63]. Koski et al. [63] analyzed the impacts of molecular weight on the electrospun polyvinyl alcohol (PVA) fiber morphologies while in fact, the molecular weight plays an important role that affects the nanofiber morphologies. At 25 wt.%, at a low molecular weight (9000–10,000 g/mol), beads were present on the nanofiber. At an intermediate molecular weight (13,000–23,000 g/mol), nanofibers formed in a uniform structure and beads were absent whilst high molecular weights (31,000–50,000 g/mol) resulted in flat-shaped nanofibers. As the concentration of the solution increased, the diameter of the fibers and the distance between them expanded, resulting in a gradual transition from circular-shaped to flat-shaped fibers. Gelb et al. [64] investigated the effects of the polymer solution properties on the electrospun nanofiber properties for drug delivery. Greater applied voltages were needed for PVA solutions with increasing surface tension to produce a consistent Taylor cone. Their research confirmed that to pull the solution into a nanoscale jet, it would take more force if the surface tension was higher, thus explaining that surface tension plays a large role in the spinnability of an electrospinning process.

Raksa et al. [65] studied the silk fibroin (SF) incorporated PVA nanofibers’ shape and mechanical characteristics, which were affected by humidity during electrospinning. The fiber’s shape and thickness became more irregular as the relative humidity rose. The fiber diameter became smaller when the relative humidity became higher. At a relative humidity of 80%, the SF/PVA nanofibers exhibited a smooth morphology and beads were absent on the nanofiber. Meanwhile, the distance of the interconnecting pores showed a decrement as the humidity rose. Yang et al. [66] investigated the impacts of the working temperature on the fabrication of the electrospun nanofiber in which this parameter obviously plays an important role in electrospinning. The smooth surface of the nanofiber was generated as the working temperature increased. As the working temperature was increased from 20 to 60 °C, the PAN nanofibers shrank in size. However, when the working temperature was raised to 80 °C, the resulting PAN nanofibers had an average diameter of 260 ± 40 nm, indicating that the temperature increment from 60 to 80 °C had no effect on the creation of PAN nanofibers. Table 1 shows some of the possible factors influencing the production of nanofibers.

A variety of synthetic and natural polymers have been employed in the construction of nanofibrous scaffolds with a variety of structural characteristics. Synthetic polymers, as opposed to natural polymers, often offer greater versatility in terms of production, processing, and alteration as well as being more cost-effective than natural polymers. It is also important to note that their mechanical characteristics may be modified efficiently and selectively. Synthetic polymers, however, have poor bioactivity and hence need more alterations than natural polymers. Natural polymers, in contrast, are intrinsically bioactive, exhibiting cell-interactive domains on their backbones, and scaffolds created from them promote greater cell attachment, multiplication, and differentiation than scaffolds generated from synthetic polymers [69]. Blends of different polymers have been used instead of single polymers to obtain the desired properties. This is because blends combine the benefits of different polymers and get around their weaknesses. For example, cress seed oil enhanced the polymer compatibility and modified the viscosity behavior of a polymer mixture of polyvinyl alcohol and starch [70]. Moreover, to utilize the benefits of both synthetic and natural polymers, researchers have developed hybrid scaffolds that have physical qualities and strong bioactivity, making them particularly well-suited for tissue regeneration [71]. In another example, coaxial fibers were created using a combination method of hydrophilic polyvinylpyrrolidone (core) and hydrophobic poly(3-hydroxybutyric acid-co-3-hydroxyvaleric acid) (sheath) [72]. These fibers have the ability to optimize the release of a poorly water-soluble drug, curcumin. This combination can prolong the curcumin release up to 24 h, which significantly enhances the therapeutic effectiveness of curcumin. Table 2 lists some of the examples of natural and synthetic polymers that have been electrospun to form nanofibers.

### 2.1. Nanofiber for Drug Delivery

In order to produce the intended therapeutic effect, a medication must be administered via the proper drug delivery system, which ensures that the drug’s precise release profile is maintained. The location, duration, and rate of release of a medicine must be tailored to the therapeutic target of the drug to the greatest extent feasible. Unfortunately, when it comes to the design of medication delivery systems, this might be a significant obstacle. Of the numerous ways to create a nanofibrous membrane, electrospinning is one that seems to hold promise since it allows for medications to be put into the nanofibrous membrane and their release at varying periods can be regulated [94]. Electrospinning has proven to be a simple and beneficial method used to produce, from the micrometer to nano scale, fiber materials for implementation in tissue regeneration, drug carriers, and wound dressing [94,95,96]. In this way, they are among the most general and promising drug delivery systems, and they may be tailored to a broad variety of drug-release kinetics when used in combination with other drugs [94]. Nanofibers may be used to produce instantaneous and controlled medication release in a variety of situations [97,98].

The fabrication of electrospun fibrous scaffolds follows a distinct hierarchy based on a range of geometrically controlled approaches. Dual extrusion electrospinning is a method to create a multi-layered 3D scaffold by layering the fibrous meshes of two different feed materials in an alternate way to make micro/nanomixed meshes. Remarkably, employing lysozyme as the model medication and poly(vinylpyrrolidone)/Eudragit^®^ RS100 as the film forming polymers, Edmans and colleagues successfully created a dual-layer mucoadhesive patch via an ethanol/water combination by applying the dual extrusion electrospinning approach for protein delivery to the oral mucosa [99]. Melt electrospinning is a type of electrospinning that is driven by temperature and uses a higher temperature. This method uses a polymer melt instead of a polymer solution so that 3D scaffolds can be made with the highest level of control over their porosity and alignment. In order to do this, the polymer is put into a syringe, heated to an appropriate high temperature, and pumped with air pressure. This method is better because it avoids using most of the harmful solvents. For instance, melt electrospinning was employed to deposit PCL loaded paclitaxel. During the process, the jet shot straight to the collector while whipping (compressed jet) happened close to the collector, creating random-placed fibers. The drug–polymer solution was heated to a 72 °C heating temperature and 150 kPa N_2_ gas pressure was used during the melt electrospinning process [100]. The dual-spinneret system based on melt electrospinning provided a novel technique for tailoring a high functional scaffold for drug delivery. In addition, ultrasound mediated electrospinning is an innovative electrospinning method used in the manufacturing of nanofibers. This method was the latest method that Laidmäe et al. patented in 2016 [101]. It uses targeted ultrasound bursts of high intensity to form an ultrasonic fountain on the interface of the polymeric solution. Around the top of the fountain, an electric field initiates the formation of a Taylor cone, through which a nanofiber jet is ejected [102,103]. For example, Partheniadis and colleagues constructed a polyethylene oxide loaded theophylline nanofiber using conventional electrospinning and ultrasound enhanced electrospinning. During the process, the drug–polymer solution was put in a positively charged vessel that was in direct contact with the Mylar membrane while a negatively charged collector plate was put over the ultrasonic fountain [104].

An appropriate technique of drug loading should be developed in order to achieve the optimal drug release kinetics, taking into consideration the characteristics of the drug to be administered. Before conducting electrospinning, drugs may be simply dissolved or disseminated in the polymer solution, or in rare situations, they can be physically or chemically bonded to the nanofiber surface [105,106]. Primarily, the nanofiber composition and interior structure are critical to achieving the desired medication release patterns.

Many medications have therapeutic implications that are dependent on their ability to produce rapid effects. As a result, the dosage form must be developed so that the drug is released immediately, or as rapidly as feasible, following administration. Many medicines with quick pharmacological activities are insoluble in water and hence have poor disintegration [106]. Thus, to ensure prompt drug release, such drugs need to be stored in a manner that provides fast wetting and breakdown. Hence, nanofibers are a well-studied and promising delivery technique for poorly soluble medicines. The identification of a suitable water-soluble polymer as the nanofiber-matrix to protect the drug in a noncrystalline condition and that allows for fast wetting, breakdown, and drug dissolution is vital. Table 3 shows a list of such polymers. The primary characteristics that form nanofibers and makes them attractive candidates for achieving immediate drug release are their high specific surface area ratio, which constitute a significant area of contact for dissolution, their high porosity, and their ability to convert crystalline drugs to an amorphous form [107,108]. Moreover, if an appropriate water-soluble polymer is employed for the inclusion of a medication into nanofibers, the dissolution profile and solubility properties of the drug as well as its bioavailability may be increased significantly [109,110].

In contrast to the immediate drug release mechanism, modified-release techniques are intended to accomplish the required pharmacological effects by extending or delaying drug delivery or by targeting a particular region inside the body. Prolonged-release drugs are designed to keep the medication accessible for an extended length of time after intake. This enables a decrease in the number of doses required in comparison to a medicine delivered in a traditional dosage form [121]. Prolonged drug release is also referred to as ‘controlled release’, ‘extended release’, and ‘sustained release’ [122]. In order to provide prolonged drug release, nanofibers made of biodegradable or swellable polymers that breakdown gradually and in a regulated way and that swell in a biological environment are very desirable choices [123,124]. An important factor in long-term drug release from a nanofiber mat is its hydrophobicity and the thickness of the nanofibers [125]. It is possible to extend drug release by using core-shell nanofibers, which have numerous drug-loaded layers, or an outer polymer layer that acts as a rate-controlling barrier [126]. In this paper, for sustained drug release, nanofibers were categorized into two types based on their structural features. The first prolonged drug release was based on matrix-type nanofibers, which were composed of drug and polymer blends [127], whilst the other type was core-shell nanofibers, which were either multi-matrix systems with multiple drug-loaded layers [128] or reservoir-type systems [129], in which the outermost part acts as a barrier to drug release. Table 4 shows the examples of a prolonged drug release system.

Stimulus-responsive polymers may be used as a basic matrix-type nanofiber construction or as a core-shell nanofiber layer to generate stimulus-activated drug release in environments such as pH [136], water [137], CO_2_ responsive [138], and electroresponsive [139]. After being exposed to an appropriate stimulus, responsive polymers can display changes in their physicochemical characteristics. A burst release is followed by a persistent release in a biphasic drug release system [140]. In addition, a simple matrix and core-shell nanofiber construction can be utilized for biphasic drug release [141,142]. Table 5 displays the examples of stimulus-responsive drug release polymers and biphasic drug release polymers.

### 2.2. Types of Nanofiber Composite Used in Drug Delivery

**Figure 4:**
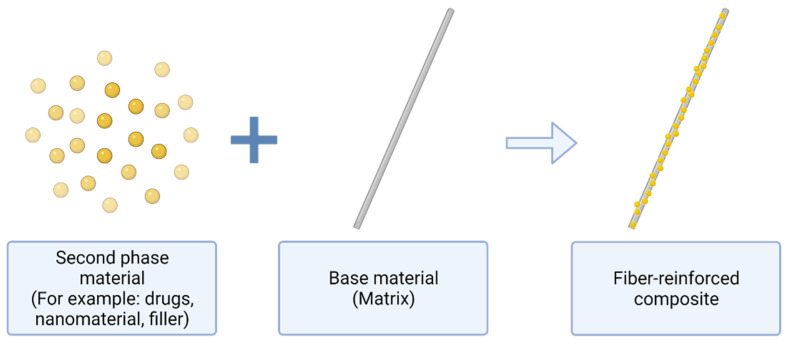
An illustration of the filler material integrated with the base material producing a reinforced composite.

Composite materials are materials that are anisotropic and inhomogeneous in nature. A composite material is created by mixing a minimum of two or more natural or synthetic components, frequently with contrasting physical or chemical properties, to form a new stronger material [155]. However, the component elements do not entirely mix together or lose their unique identities; rather, they integrate and offer their most beneficial characteristics in order to enhance the ultimate result or final product. Composite materials are classed according to their composition, which is divided into two categories: base material and filler material [156]. When it comes to structures, the base material, which binds or keeps the filler material together, is referred to as a matrix or a binder material, whilst the filler material may be found in the presence of natural or synthetic materials in the shape of sheets, fragments, particles, fibers, or filaments [156]. As presented in Figure 4, fiber is an example of a filler material being modified with a base material, producing fiber-reinforced composites.

Nanofiber composites may be roughly divided into three categories according to the matrix component: polymer matrix composites (for example, polyvinyl alcohol/carbon nanotubes nanofibers [157]), ceramic matrix composites (for example, collagen/nano-hydroxyapatite nanofibers [158]), and metal matrix composites (for example, zinc oxide/polyacrylonitrile nanofibers [159]). Polymer matrix composites are composed of a continuous phase of various organic polymers and a dispersed phase of reinforced fibers [160]. The continuous phase acts as a matrix, holding the fibers together and allowing for effective weight transmission between them [161,162]. Ceramic matrix composites are typically made up of ceramic fibers embedded in a ceramic matrix in which the fabrications are intended to alleviate the major disadvantage of inflexible ceramics, particularly, their brittleness [163]. Metal matrix composites are materials that involve the addition of a reinforcement to a metal or alloy matrix in particle form, fibers, whiskers, or even a sheet metal [164]. Metal matrix composites have a diverse array of characteristics that significantly outperform rigid parent materials such as good mechanical properties, resistant to wear, and corrosion [164].

Generally, a suitable composite is chosen based on the application site where it will be employed. Ceramic–polymer nanofiber composites may be an excellent alternative for osteogenic applications where inorganic–organic components play a significant role in the bone tissue structure. For example, hydroxyapatite/polycaprolactone nanofibers have been used as a drug carrier for rifampicin for orthopedic implant related infections [165]. Hydroxyapatite was used because it is a biocompatible osteoconductive ceramic that has been shown to be an important material in improving bioactivity [165]. Furthermore, polymer–polymer nanofiber composites may be ideal for use in soft tissue repair such as the skin or heart. For instance, a biocompatible patch for cardiac tissue engineering constructed of a hydrophilic intermediate layer made of a combination of silk fibrin and polyvinyl alcohol, while the upper and lower layers were developed from polycaprolactone and polylactic acid individually [166]. As a result, the material used was solely determined by the functional attributes necessary for the particular application.

### 2.3. Characteristics of the Nanofiber Composite

**Figure 5:**
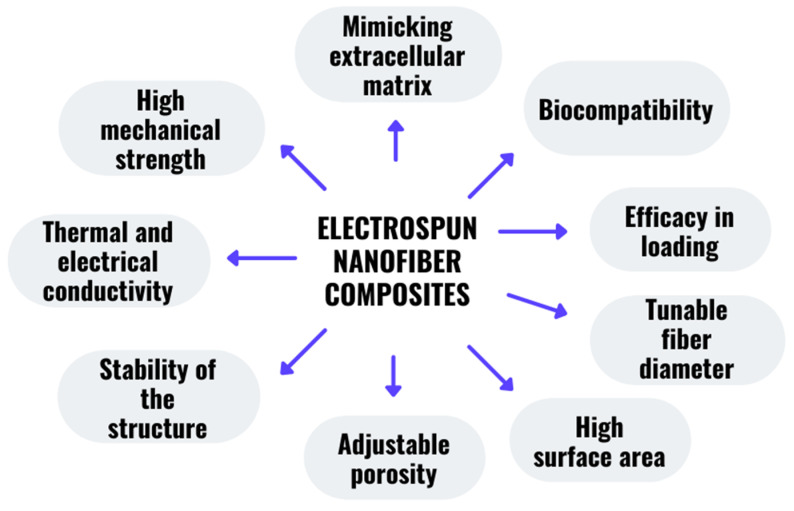
The important characteristics of the nanofiber composite.

Nanofiber composites have been shown to have a considerably greater surface area than common composites while maintaining their volume portion [167]. Because the increased surface area compensates for the poor bonding between the fiber matrix interphase, nanofiber composites are stronger than conventional composites made with the same volume percentage [156]. Surface treatments might be applied to these composite structures to improve or add new beneficial applications. For instance, the electrospinning process was used to create novel core-shell nanofibers for the encapsulation of vancomycin in the shell section, which was made of polyethylene oxide/chitosan in the shell while polyvinylpyrrolidone/gelatin encapsulated imipenem/cilastatin in the core sections [168]. This study revealed that incorporating imipenem/cilastatin into the core portion played a part in a slower and more regulated release than a faster release of vancomycin in the shell section [168]. The mechanical strength of the constructed core-shell nanofiber had an ideal mechanical strength to be utilized in biomedical applications [168]. A few of the most important properties of the nanofiber composites are illustrated in Figure 5. These characteristics are quite adaptable and may be tailored to meet individual requirements and applications. For instance, the impact of various solvents and solvent binary combination on the morphology of pullulan nanofibers [169]. Primarily, the shape and sizes of the nanofibers were linked to the solution viscosities, solvent–polymer interactions, and solvent vapor pressure [169].

## 3. The Use of Nanofiber Composite as a Drug Delivery System

**Figure 6:**
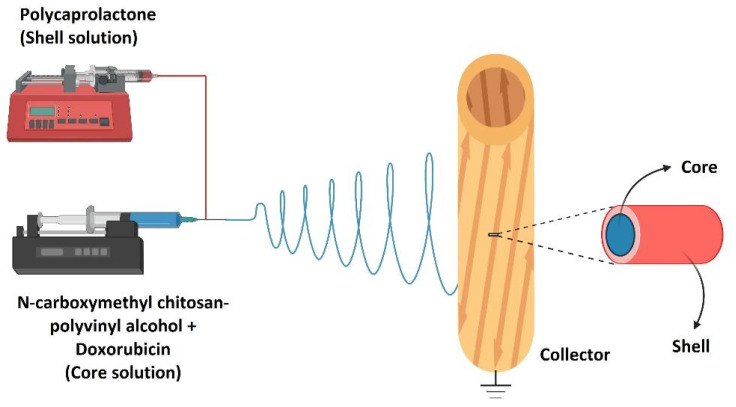
An illustration of the coaxial electrospinning setup.

A growing number of researchers are interested in the unusual physiochemical features such as the huge surface area, smaller diameter, and high aspect ratio of the composite nanofibers made from biodegradable and biocompatible polymers [170,171,172,173]. An electrospun nanofiber that meets these criteria is ideal for use as a drug carrier. Composite nanofibers refer to multiphase fiber structures in which minimally, one of the phases has a dimension in the nanoscale. Primarily, the mechanical characteristics, heat resistance, chemical stability, surface and optical properties, electrical conductivity, and molecular permeability of the composite nanofibers outperformed those of the separate material components in a variety of areas. The potential of electrospun nanofibers being incorporated with a composite to enhance the properties can be seen in a study conducted by Rezk et al., who incorporated beta-tricalcium phosphate into polycaprolactone and cellulose acetate to form a composite mat to imitate apatite to stimulate the biomineralization process [174]. They also loaded simvastatin into a multi-membrane of polyvinyl alcohol and polyvinyl acetate to promote and enhance the osteogenic process with the use of controlled drug release [174]. Li et al. [175] successfully fabricated a compound nanofiber made of flexible inorganic composites with carboxy modification for sustained drug release. This study revealed that a greater amount of drug loading capacity, and a slower drug release rate, were achieved once these nanofibers were further treated with carboxyl radicals [175]. The ionic contact involving daunorubicin molecules and the carboxyl group, which has been confirmed by FTIR, was the primary mechanism of the improved drug loading [175]. Abasalta et al. [176] performed a coaxial electrospinning method to produce core-shell nanofibers composed of an *N*-carboxymethyl chitosan-polyvinyl alcohol/polycaprolactone composite loaded doxorubicin, an anticancer drug. The incorporation of *N*-carboxymethyl chitosan into the polyvinyl alcohol solution was then electrospun together separately with polycaprolactone through the coaxial electrospinning setup, as shown in Figure 6 [176]. In contrast to physiological pH, the carboxylic and amine groups of *N*-carboxymethyl chitosan were shown to be weak at a pH of 5.5, resulting in greater swelling and quicker release of doxorubicin from the nanofibers at acidic pH [176]. Doxorubicin molecules were more easily dispersed from the nanofibrous matrix at acidic pH because of the increased solubility of doxorubicin at acidic pH [176]. Consequently, the composite nanofibers that were constructed from the core-shell matrix are ideal candidates for use as a pH-sensitive drug carrier for doxorubicin. Zhao et al. [177] successfully constructed a drug delivery system from a composite nanofiber made of carboxymethylation curdlan incorporated polyethylene oxide through the electrospinning process. In their research, the presence of carboxymethylation curdlan in polyethylene oxide can increase the conductivity of the spinning solution, which was due to the enhanced ionization properties of carboxymethylation curdlan [177]. The elongation of the nanofibers dropped notably when the carboxymethylation curdlan concentration in the nanofibers was increased [177]. The presence of hydrogen bond interactions between the carboxymethylation curdlan and polyethylene oxide in the nanofibers resulted in the tensile strength and Young’s modulus being notably escalated as the carboxymethylation curdlan concentration increased [177].

### 3.1. Applications in Pharmaceuticals

The construction of nanofiber composites as a possible drug delivery system for a variety of medicinal purposes has been intensively investigated. Most of the medications have low solubility, stability, and low biodistribution within the biological system. Aside from these challenges these medications do not have exact targeting abilities and they also have a short half-life, which further leads to systemic toxicity and rapid removal. Amer et al. [178] developed a composite nanofiber made of polyvinyl alcohol incorporated with quercetin and essential oils for acne alleviation. Quercetin was used in this study as it contains antioxidant, anti-inflammatory, anti-cancerous, and anti-bacterial properties [178]. In this research, they demonstrated that this composite nanofiber promoted an acceptable skin deposition, substantially more antibacterial activity against Propionibacterium acne than quercetin alone, and was completely safe on the skin fibroblastic cells [178]. Clinical testing on acne patients revealed that the nanofibers reduced inflammatory, comedonal, and total acne lesions by 61.2%, 14.7%, and 52.9%, respectively, indicating a possible composite nanofiber working as a drug carrier to treat skin diseases [178]. Pourpirali et al. [179] constructed an electrospun composite nanofiber made of polycaprolactone/gelatin encapsulated titanium dioxide nanoparticles and metformin-loaded mesoporous silica nanoparticles using electrospinning. The incorporation of titanium dioxide nanoparticles and metformin-loaded mesoporous silica nanoparticles into hybrid polymeric nanofibers improved the mechanical characteristics and decreased the burst release of metformin, resulting in a three-week continuous release [179]. Additionally, after 28 days of culture, the created composite scaffold successfully increased the viability and proliferation rate of human adipose-derived stem cells [179]. As a matter of course, these findings indicate that a composite nanoplatform may offer potential benefits for obtaining adequate amounts of functional human adipose-derived stem cells and enhancing scaffold-based regenerative treatments [179]. Successful stem cell treatments must develop innovative expansion procedures for adipose-derived stem cells that sustain the cells’ multipotency, even after extensive cell expansions. Thus, Mohebian et al. [180] developed a nanofiber composed of curcumin-loaded mesoporous silica nanoparticles incorporated into polycaprolactone/gelatin using the electrospinning method. The in vitro drug release study results demonstrated that the mesoporous silica nanoparticles inserted into the electrospun nanofibers permitted for prolonged curcumin release, which may have a beneficial potential to increase the lifetime and long-term proliferation of human adipose-derived stem cells without diminishing their stemness potency and undergoing cellular senescence [180]. Electrospun nanofibers are indeed a very appealing material that may be employed as a foundation for the formation of multiple-drug dosage. Chi et al. [181] conducted an analysis on non-steroidal anti-inflammatory drugs: paracetamol, nimesulide, and ibuprofen loaded into polyvinylpyrrolidone/polycaprolactone composite nanofibers. They conducted high-speed capillary electrophoresis separation and detection at 200 nm to evaluate the multiple medicines emitted from the polyvinylpyrrolidone and polycaprolactone composite nanofibers [181]. Since polyvinylpyrrolidone is a hydrophilic polymer, its increment in the polyvinylpyrrolidone/polycaprolactone composite ratio boosts the release of medicines inside the nanofiber in a dissolution medium as well as improves the dissolution efficiency [181]. Tort et al. [144] successfully constructed an effective pramipexole-loaded nanofiber for use as a floating drug delivery system embedded in cast films made of polyethylene oxide and sodium bicarbonate. The floating nanofiber membranes were composed of polymer hosts Eudragit RL and RS, which were fabricated using the electrospinning technique [144]. They found that adjusting the Eudragit RS/RL ratio is an easy way to modify the integrated pramipexole’s release kinetics [144]. According to the present study, the polyethylene oxide/sodium bicarbonate film integrated in an electrospun nanofiber-based floating gastro-retentive drug carrier delivered a 24 h release of pramipexole [144].

### 3.2. Tissue Engineering

Tissue engineering is a part of regenerative medicine, which is a multidisciplinary discipline. Proposing new methods to keep tissues and organs functioning correctly, particularly after they have been damaged, is its primary goal [182]. Despite the fact that transplanted organs may be used to cure patients with sick and damaged organs, there is indeed a major scarcity of donor organs that worsens year after year as the elderly population grows [183]. Tissue engineering relies heavily on nanomaterials such as nanofibers. Basu et al. [184] proved that by using the electrospinning method, they were capable of producing a scaffold for soft tissue engineering applications that are composed of polyethylene oxide and carboxymethyl cellulose/polyethylene oxide scaffolds. In this research, the incorporation of polyelectrolyte carboxymethyl cellulose with polyethylene oxide generated nanofibers with smaller diameters compared to the pure polyethylene oxide nanofiber. Cell viability was confirmed after 24 h of culture, and the thiazolyl blue tetrazolium blue (MTT) test findings indicated that the scaffolds may be able to support cell proliferation and metabolic activities [184]. This integration can be further studied to form a nanofibrous drug delivery carrier.

In another study conducted by Bazzi et al. [185], a hybrid nanocomposite nanofiber comprised of a chitosan-polyvinyl alcohol matrix reinforced with graphene nanoplatelet fillers was also fabricated by the electrospinning technique. The addition of 1% of graphene nanoplatelet fillers into the matrix reduced the diameter of the nanofibers as a result of the conductivity enhancement of the chitosan-polyvinyl alcohol suspension, while at the same time that these findings were made, this nanofiber promoted the improvement in the cell activity including growth, proliferation, and migration [185]. Due to the hydrogen bonding among elements of the nanocomposite fibers and the electrostatic interaction that occurs among them, graphene nanoplatelets are distributed throughout the fibers in a uniform manner, resulting in enhanced mechanical characteristics [185]. Thus, these characteristics offer this nanocomposite an excellent chance as a drug delivery candidate for use in tissue engineering applications.

Developing biomimetic scaffolds that mirror the structure and biological features of the natural extracellular matrix is a crucial necessity for the treatment of injuries and illnesses via tissue engineering. Nitti et al. [186] used electrospinning to produce a possibly idyllic scaffold for tissue engineering applications composed of an aligned chitosan nanofibrous mat treated with amino acids and L-arginine as a stabilizing agent. According to the present study, they found that nanofiber mats with L-arginine had better wettability and architectural stability compared to the untreated chitosan nanofiber mat [186]. After 60 days of being immersed in tris(hydroxymethyl)amino-methane hydrochloride, the pristine nanofiber mats turned out to have a significantly degraded nanofiber structure [186]. On the other hand, the L-arginine treatment on the chitosan nanofiber mat was shown to preserve the nanofibrous structure [186]. The biomimicking capability of the nanofibrous structures to be utilized for soft tissue regeneration was further demonstrated in cell-based studies employing murine fibroblasts in which the addition of L-arginine was shown to play a vital role as a chemical stabilizer and as a naturally occurring metabolic substrate for influencing cell–material interactions [186].

#### 3.2.1. Bone Tissue Engineering

**Figure 7:**
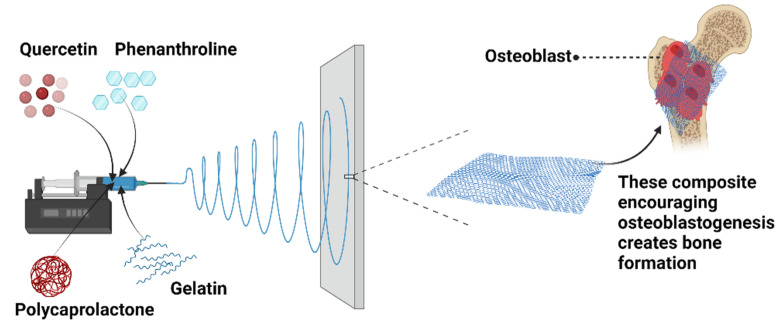
An illustration of quercetin/phenanthroline incorporated with polycaprolactone/gelatin.

Tissue engineering approaches can be used to create bone-compatible scaffolds but creating a scaffold with significantly bioactive molecules to govern bone remodeling remains a monumental effort. Because of its three-dimensional porous nature, the electrospinning technology has been employed to create fibrous scaffolds for biological purposes. Electrospun scaffolds have a very high surface-to-volume ratio, pore diameters ranging from a few to tens of micrometers, and tunable high porosity, which makes nanofibers as ideal constructions that are able to mimic the natural nanostructure of bone [187]. Jahanmard et al. [188] recently proved that the integration of COOH-Multiwall carbon nanotubes into polycaprolactone nanofibers was discovered to be an excellent strategy for independently controlling the material surface nanoroughness and stiffness, two critical factors associated with cell function regulation. High interfacial nanoroughness or stiffness resulted in increased osteoblast differentiation [188]. In another study conducted by Raj Preeth et al. [189] whereby they created a bioactive zinc, quercetin/phenanthroline was incorporated with polycaprolactone/gelatin to be electrospun to form nanofiber scaffolds to improve bone tissue regeneration. In addition to their osteogenic function, these composites stimulated angiogenesis in ovo. Figure 7 illustrates the incorporation of quercetin/phenanthroline with polycaprolactone/gelatin to form electrospun nanofiber composites that have been proven to enhance osteoblastogenesis for bone development.

In the meantime, Gong et al. [190] utilized icariin, a traditional Chinese medicine *herba epimedium* to be integrated into polycaprolactone/gelatin nanofibers through electrospinning to produce a novel artificial periosteum. The introduction of icariin definitely contributes to the membranes’ hydrophilicity while also promoting preosteoblast differentiation and proliferation [190]. Bakhsheshi-Rad et al. [191] constructed bone regenerating electrospun nanofibers using electrospinning, which constituted gelatin-ciprofloxacin nanofibers on the surface of a magnesium-calcium alloy. A gelatin-ciprofloxacin nanofiber coating resulted in prolonged drug release, with an initial fast drug release of roughly 20–22% within 12 h, followed by a delayed release stage that may successfully manage the infection [191]. Adding ciprofloxacin into gelatin nanofibers as a coating considerably improved the antibacterial activity and resistance to corrosion of the untreated magnesium-calcium alloy without impairing cytocompatibility [191].

#### 3.2.2. Nerve Tissue Engineering

The adult human neurological system’s potential to regenerate is frequently restricted. Consequently, individuals with nervous system impairments or trauma frequently have sensory or motor dysfunction as well as neuropathic symptoms [192]. Electrospun biodegradable nanofibers constitute a new class of potential scaffolds for nerve regeneration. The biological scaffold material, seed cells, and different growth agents are the three components of peripheral nerve tissue engineering [193]. The use of biocompatible polymer nanofibrous conduits with the regulated delivery of drugs for peripheral nerve restoration has recently received a lot of interest.

Fallah-Darrechi et al. [194] constructed a conductive conduit from electrospun poly (ւ-lactide-*co*-D, ւ-lactide) (PLDLLA) nanofibers integrated with multi-walled carbon nanotubes (MWCNT) and 4-aminopyridine (4-AP) deposited molecularly imprinted poly(methacrylic acid) (MIP_6/4-AP_) nanoparticles. Once the MIP_6/4-AP_ nanoparticles with the maximum drug adsorption were obtained, they were then integrated with PLDLLA/MWCNT, producing an electrical conductivity of 2870 × 10^−7^ Scm^−1^ [194]. Drug release studies of the composite nanofibers showed that the presence of the PLDLLA/MWCNT nanofiber could suitably extend the 4-AP release with a gradual slope in which the emergence of the peak stage was delayed by about 12.5% until a 4 day time frame, particularly during the final hours of its release [194]. When compared to plain PLDLLA nanofibers, the culture results of adrenal phaeochromocytoma (PC12) as a neuroblastoma cell line on the ideal PLDLLA/MWCNTs/MIP_4-AP_ nanofibrous sample demonstrated the maximum cell growth without cytotoxicity. In another study, conductive nanofiber scaffolds composed of chitosan/collagen/polyethylene oxide integrated with polypyrrole were formed using electrospinning, producing a maximum electrical conductivity of 164.274 × 10^−3^ Sm^−1^ [195]. The electrical conductivity of the fabricated nanofibers was evaluated to be in the range of semiconductive materials and conductive polymers for nerve tissue application [195]. The addition of a polypyrrole polymer chain in conductive scaffolds improved the cell adhesion, growth, and proliferation [195,196]. Moreover, Mohamady Hussein et al. [197] constructed a core-shell electrospun nanofibrous membrane for a dual-drug delivery system. Phenytoin was filled into the shell layer of polycaprolactone while silver-chitosan nanoparticles were inserted into the polyvinyl alcohol core compartment of the nanofibrous membrane [197]. The addition of silver-chitosan nanoparticles into the coaxial electrospun nanofiber enhanced the cumulative phenytoin release mechanism by over half, which was 53.8% of the originally loaded phenytoin being released gradually and regulated from the matrix after 7 days [197]. The inclusion of silver-chitosan nanoparticles into the core-shell nanofiber made it more hydrophobic in the environment, enabling phenytoin to be progressively released, which makes this membrane ideal for nerve regeneration [197].

#### 3.2.3. Periodontal Tissue Engineering

**Figure 8:**
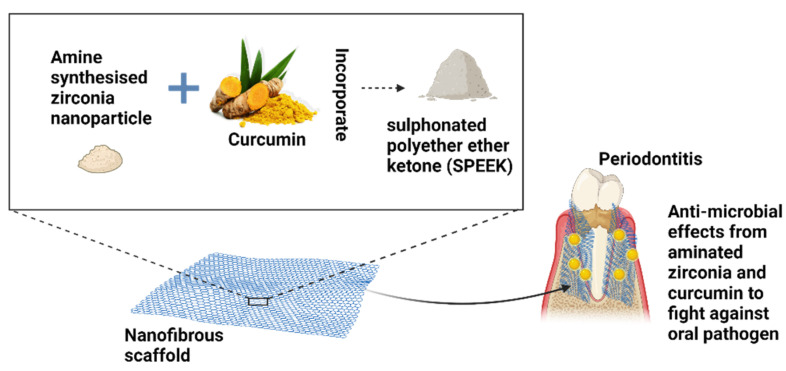
A schematic diagram showing how the nanofibrous membrane helps to fight oral pathogens with composite properties.

Periodontitis is a severe inflammatory condition that can lead to the deterioration of the periodontium and, eventually, tooth loss. Periodontal tissue is indeed a unique component of the body in which soft, mineralized connective and epithelial tissues are arranged to produce a dentogingival junction [198]. Abdelaziz et al. [199] efficaciously constructed a novel electrospun polylactic acid/cellulose aetate and polycaprolactone nanofiber integrated with hydroxyapatite nanoparticles and green-synthesized silver nanoparticle scaffolds to enhance antibacterial activity for directed periodontal tissue and bone regeneration. Nanofibers loaded with green-synthesized silver nanoparticles demonstrated inhibition of bacteria growth [199]. In vitro experiments revealed that the presence of hydroxyapatite nanoparticles increased the cell viability by roughly 50% for both types of nanofibrous scaffolds, while the addition of 10% hydroxyapatite nanoparticles also increased the tensile characteristics [199]. In addition, to accommodate periodontal regeneration, Ekambaram et al. [200] magnificently constructed innovative amine synthesized zirconia nanoparticle filled curcumin integrated sulfonated polyether ether ketone (SPEEK) nanofibrous scaffolds. Curcumin significantly identified anti-bacterial properties, augmenting its benefit in the treatment of periodontitis [200]. Amine, zirconia, and curcumin were added to the nanofibers to increase the physicochemical, mechanical, and biological properties of the nanofiber scaffold, which are suitable for periodontal regeneration purposes [200]. Schematic 8 demonstrates the comprehensive role of the constructed electrospun amine functionalized zirconia and curcumin incorporated SPEEK nanofibrous membrane to combat oral pathogens through the delivery of the anti-microbial properties of amined zirconia and curcumin Figure 8.

### 3.3. Wound Dressing

The skin is the body’s biggest organ, and it serves as the body’s principal defensive mechanism, preventing disease infiltration. A wound is a form of injury in which the dermis has been damaged by an abrasion, laceration, puncture, and avulsion. With the rising expenses of wound care, several studies have been conducted to investigate new medicines that might reduce the inflammation, particularly in infected wounds [201,202].

**Figure 9:**
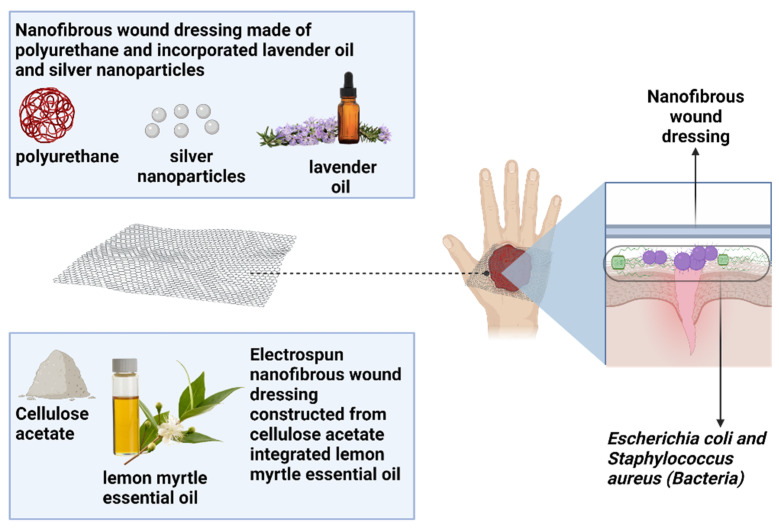
A schematic diagram of how essential oil incorporated with polymer works on combating *Escherichia coli* and *Staphylococcus aureus*.

Sofi et al. [203] conducted an in vitro investigation that showed that nanofibrous dressings made of polyurethane and incorporating lavender oil and silver nanoparticles has combinatorial antibacterial effects against *Escherichia coli* and *Staphylococcus aureus*. The hydrophobicity of the polyurethane fibers was altered by the diffusion and penetration of lavender oil into the nanofibers [203]. Additionally, due to the strong hydrophobicity of polyurethane nanofibers encompassed with lavender oil and silver nanoparticles, cell fixation research indicates that fibroblasts grew in their natural shape on the fiber mats compared to the spherical shape on the pristine nanofibers [203]. Another study using an essential oil as a natural antibacterial agent for wound healing was conducted by Beikzadeh et al. [204], where they utilized lemon myrtle essential oil (LMEO) encapsulated in cellulose acetate electrospun nanofibers. At the minimum LMEO loading concentration, the LMEO-loaded cellulose acetate electrospun nanofibers proved to have completely eradicated *Escherichia coli* and *Staphylococcus aureus* [204]. The electrospun fiber mats with modest LMEO loading demonstrated continuous LMEO release over a long period of time, and the nanofibers kept their strong antibacterial capabilities, even after two storage periods, making these nanofibers suitable as wound dressings [204]. Figure 9 illustrates the functional nanofibrous wound dressing composed of essential oil to fight against bacteria.

Lan et al. [205] constructed a low cytotoxicity coaxial electropsun nanofiber membrane acting as an antioxidant and antibacterial wound dressing treatment. Antioxidant tea polyphenols were combined with a polyvinyl alcohol membrane while antibacterial ε-poly(L-lysine) was integrated with polycaprolactone as a shell [205]. ε-Poly(L-lysine) demonstrated a rapid release to inhibit bacterial growth in the early stages, whereas tea polyphenols demonstrated a controlled release to eliminate excess reactive oxygen species (ROS) [205]. Acute injuries necessitate the rapid release of a medicine to combat infections without causing any negative effects [206]. Qiu et al. [196] successfully merged photodynamic antimicrobial chemotherapy (PACT) with electrospinning for wound healing purposes. Indocyanine green (ICG) as a photosensitizer was incorporated with chitosan/polyvinyl alcohol to form an electrospun nanofiber membrane in which in vitro, ICG exhibited good antibacterial properties against methicillin-resistant *Staphylococcus aureus* (MRSA) and meropenem-resistant *Pseudomonas aeruginosa* (MRPA) [196]. ICG is released from nanofibers in vivo to destroy germs on the injury surface and inhibit infection [207].

### 3.4. Cancer Therapeutics Drug Delivery System

Chemotherapy using anticancer medications is the most often used treatment for cancer, however, it frequently fails due to the toxic impact of the chemotherapeutic agents, which have been found to have severe adverse effects. As a result, major efforts are being put toward the development of an advanced drug delivery system that can specifically target malignant areas while causing minimum adverse effects in other sections of the body. Bazzazzadeh et al. [208] effectively assembled magnetic MIL-53 nanometal organic framework particles being combined with poly(acrylic acid) grafted-chitosan/polyurethane core-shell nanofibers for the sustained delivery of temozolomide (TMZ) and paclitaxel (PTX) against glioblastoma cancer cells. The encapsulation effectiveness of TMZ and PTX for synthesized core-shell nanofibers was more than 80%, indicating that core-shell fibers have a significant potential for use in drug carriers [208]. Temperature and pH each plays a vital role in releasing PTX and TMZ in the core-shell nanofibers; the minimum drug release at a pH and temperature of 7.4 and 37 °C, respectively, while the maximum drug release at a pH and temperature of 5.5 and 43 °C individually [208]. Flow cytometry revealed that 31.3% and 49.6% of glioblastoma cancer cells experienced apoptotic cell death when exposed with a magnetic MIL-53 nanometal organic framework particle/poly(acrylic acid) grafted-chitosan/polyurethane loaded TMZ and PTX in the non-existence and existence of alternating magnetic field, respectively [208]. Arumugam et al. [198] developed an anticancer composite nanofiber made of silk fibroin/cellulose acetate/gold-silver nanoparticles. In this study, the composite nanofiber inhibited most of the human breast cancer [209]. In another study on the breast cancer treatment accomplished by Mohebian et al. [210], they modified curcumin (CUR) as a natural anticancer agent inside mesoporous silica nanoparticles (MSNs), then electrospun the nanocomposite with poly(lactic-co-glycolic acid) (PLGA) through electrospinning, producing a controlled drug release. This study showed loading CUR/MSNs into the PLGA nanofiber produced a steady and extended drug release behavior [210]. This composite nanofiber also had greater in vitro cytotoxicity, low migration, and was capable of enhancing apoptosis induction [210]. More research on breast cancer treatment has been conducted in which CUR and PTX were encapsulated in graphene oxide, a nanocarrier, then electrospun with pullulan to form a nanofiber drug carrier [211]. The sustained release of both medications was validated in this research, and a simultaneous impact of PTX and Cur was demonstrated against breast cancer cells, where cell growth was suppressed [200].

## 4. Future Perspectives

Composite nanofiber architectures have aided in the evolution of drug delivery applications by allowing for the regulated delivery of therapeutic agents in consistent dosages over extended periods of time, cyclic dosing, and the infinitely adjustable release of both hydrophilic and hydrophobic medicines. Current drug delivery developments are now based on a fundamental construction of polymers that are suited for particular contents and are made to perform diverse biological activities. Optimizing a composite with a polymer as electrospun nanofibers to form a drug carrier to deliver the medications to a specific location is extremely crucial in drug delivery applications, tissue engineering, cancer treatment, and pharmaceutical applications. Future work may further focus on the development of a smart drug delivery system that is sensitive to optical stimulation, pressure stimulation, electric impulses, ultrasound exposure, or electromagnetism in order to provide targeted drug administration. Additionally, the fast growth of knowledge and the creation of more advanced mutual systems could help make it easier to make smart, integrated devices that can control the amount of drug released from the nanofibrous membrane when the body is stimulated. On the other hand, second phase nanomaterials, which are also known as filler materials, possess appealing characteristics such as high surface area in the nanoscale, and great biocompatibility, which produces a good drug carrier. Therefore, more research using second phase materials with natural polymers such as gum arabic, chitin, honey, pectin, wool, starch, dextran, and chitosan is needed for a controlled and targeted drug delivery system.

## 5. Conclusions

Over the last few decades, electrospinning has changed significantly. Electrospinning is a quick and easy way to make drug delivery systems that are smart and can be controlled. Electrospinning can be used in many different ways, and it is a great place to start when making new ways to deliver drugs that improve therapy while reducing the side effects. The choice of drugs and polymers can easily be adjusted for different uses or areas. By changing the mechanical properties or release kinetics, the nanofiber could lead to new ways to make precise medications. Amongst the most complex and challenging obstacles in medication delivery is getting the intended therapeutic agent to the right place at the right time with the right dosage. In this review paper, we highlighted the utilization of nanofibers as drug loaders or drug carriers for controlled drug release. The design of nanofibers is essential for drug delivery purposes, which in this review paper, we emphasized the categories of nanofiber composites being used in a drug delivery system. Characteristics of the nanofiber composites are highly customizable to specific purposes and applications. Nanofibrous scaffolds are an area of research that has not been fully explored yet in diabetes, hormone treatment, and immune disorders. The problems with electrospun nanofibers might be easier to solve with a thorough and structured plan. Enhanced scaffolds that incorporate tissue engineering with controlled drug release without negative side effects could be a useful tool in the future for treating patients in hospitals. Configurable nanofibers could play a pivotal role in personalized medicine because of their unique properties and ease of use.

## Figures and Tables

**Table 1:** The electrospinning factors that affect the fabrication of the nanofibers.

Parameters	Details	
Process parameters	Applied voltage	The formation of average diameter of nanofibers of increased with the applied voltage [50,53,54].
	Flow rate or feeding rate	Diameter of nanofibers decreases as the flow rate decreases [55,67].
	Distance from needle tip-to-metal collector	When distance tip-to-collector increases, average diameter of nanofibers decreases [59].
Solution parameters	Solution concentration	Fiber diameter decreases with solution concentration [61].
	Solution viscosity	Fiber diameter increases as solution viscosity increases [63].
	Molecular weight	Diameter of fiber increases with molecular weight [68].
	Surface tension	Electrospun fibers have a tendency to be uniform and continuous when the surface tension is low [69].
Ambient parameters	Humidity	As the humidity increases, the interconnecting pores and the diameter of the fibers decrease [65].
	Temperature	A temperature increase resulted in a decrease in the fiber diameter [66].

**Table 2:** Polymers that have been electrospun from a solution to a nanofiber.

Polymer	Solvent	Concentration	References
Cellulose acetate	Acetone: dimethylacetamide: ethanol (3:2:1 *v*/*v*)	17% (*w*/*v*)	[73]
Chitosan	Ultrapure water and 0.7% acetic acid	2% (*w*/*w*)	[74]
Ethyl cellulose	Water: ethanol: acetic acid (2:2:6 *v*/*v*/*v*)	30% (*w*/*v*)	[75]
Gelatin	Acetic acid (20% *v*/*v* in distilled water)	20% (*w*/*v*)	[76]
Fish gelatin	Distilled water	40% (*w*/*v*)	[77]
Gum Arabic	Deionized water	5.8% (*w*/*v*)	[78]
Collagen	Hexafuoroisopropanol	10% (*w*/*v*)	[79]
Pectin	Water with 2% (*v*/*v*) acetic acid	4 wt.%	[80]
Polyethylene oxide	Deionized water	4% (*w*/*v*)	[81]
Poly(D, L)-lactide-co-glycolide	Tetrahydrofuran: *N*,*N*-dimethylformamide (3:1 *v*/*v*)	25% (*w*/*v*)	[82]
Polyvinylpyrrolidone K60	Anhydrous ethanol	8%	[83]
Poly(3-hydroxybutyrate)	Trifluoroacetic acid	9 wt.%	[84]
Poly(glycerol sebacate)	Polyol glycerol/sebacic acid (1:1)	30 wt.%	[85]
Poly(l-lactic acid)	Chloroform: acetone (2:1)	25% (wt/*v*)	[86]
Poly(3-hydroxybutyric acid-co-3-hydroxyvaleric acid)	Chloroform: trifluoroethanol (3:2)	7.5% (*w*/*v*)	[87]
Poly(L-lactide-co-e-caprolactone)	1,1,1,3,3,3-hexafluoro-2-propanol	10% (*w*/*v*)	[88]
Poly(*N*-isopropylacrylamide)	Anhydrous ethanol	25% (wt/*v*)	[89]
Poly(vinylidene fluoride)	Acetone/N,*N*-dimethyl acetamide (70:30)	16 wt.%	[90]
Polyacrylonitrile	*N*,*N*-dimethylformamide		[91]
Polyamide-6	Formic acid	15 wt.%	[92]
Polycaprolactone	Acetic acid:formic acid (50:50 *v*/*v*)		[93]

**Table 3:** Some examples of the polymers used and integrated drugs or active agents for immediate drug release.

Polymer (s)	Drug or Active Agent	References
Polyethylene oxide and poloxamer 188	Lovastatin	[111]
Polyvinylpyrrolidone and Soluplus^®^	Meloxicam	[112]
Poly (lactic acid) and butylene poly (butylene adipate)-co-(butylene terephthalate)	Aceclofenac	[113]
Cellulose acetate	Alpha-arbutin	[114]
Hydroxypropyl-beta-cyclodextrin and polyvinylpyrrolidone	Acyclovir	[115]
Polyethylene oxide and poloxamer 407	Carvedilol	[116]
Polyvinylpyrrolidone and ethyl cellulose	Ciprofloxacin	[117]
Poly(lactic-co-glycolic acid) and polyvinylpyrrolidone	Pirfenidone and moxifloxacin	[118]
Polyvinylpyrrolidone and zein	Ketoprofen	[119]
Ketoprofen	1,4 trans aminohexanoic acid drug	[120]

**Table 4:** Some examples of the polymer fused drug(s) or active agent(s) for controlled drug release.

Nanofiber System Type		Polymer	Drug (s) or Active Agent (s)	References
Matrix type	Dense	Gelatin	Amphotericin B	[130]
		Poly(D,L-lactide-co-glycolide)	Ciprofloxacin hydrochloride	[98]
	Porous	Chitosan, sodium alginate, and polyvinyl alcohol	Deferoxamine	[131]
		Polyvinyl alcohol, Polyvinylpyrrolidone	5-flurouracil	[132]
		Cellulose acetate	Ferulic acid	[133]
		Polycaprolactone	Metronidazole, ciprofloxacin hydrochloride	[125]
		Polycaprolactone, parylene	Pramipexole	[134]
Core-shell		Polyvinylpyrrolidone (core), Poly lactic-*co*-glycolic acid (shell)	Pirfenidone, moxifloxacin	[118]
		Polyethylene oxide (core), Polycaprolactone (shell)	Doxorubicin hydrochloride	[135]

**Table 5:** Some of the polymer incorporated drug(s) or active agent(s) for stimulus-responsive drug release.

Drug Release Mechanism	Polymer (s)	Drug(s) or Active Agent(s)	References
pH-responsive release	Cellulose acetate, collagen	Naproxen	[143]
	Polyethylene oxide	Pramipexole	[144]
	Poly(lactic-*co*-glycolic acid)	Ibuprofen	[145]
	Polycaprolactone, gelatin	Ciprofloxacin	[146]
Mechano-responsive	Poly(vinylidene fluoride-trifluro-ethylene)	Crystal violet	[147]
Thermoresponsive	Poly(*N*-isopro-pylacrylamide-co-acrylamide-co-vinylpyrrolidone	Doxorubicin	[148]
Biphasic drug release	Polyvinylpyrrolidone, Ethyl cellulose	Ketoprofen	[149]
	Poly(butylene succinate)	Rhodamine B	[150]
	Polycaprolactone	Silver nitrate, gallium nitrate, vancomycin	[151]
	Poly(vinyl pyrrolidone), poly(vinyl alcohol)	Buprenorphine	[152]
	Polyvinylpyrrolidone, sodium dodecyl sulfate, sucralose	Helicide	[153]
Light-responsive drug release	Polyethylene glycol, poly(3-hydroxybutyrate-co-3-hydroxy valerate), cellulose nanocrystal-zinc oxide	Tetracycline hydrochloride	[154]

## Data Availability

The data presented in this study are available on request from the corresponding author.
